# Investigating genetic relationship between cancer and Alzheimer's disease

**DOI:** 10.1002/alz70855_104913

**Published:** 2025-12-24

**Authors:** Dipti Debnath, Mohammad Housini, Nicole Phillips, Robert Barber, Gita Pathak

**Affiliations:** ^1^ University of North Texas Health Science Center, Fort Worth, TX, USA; ^2^ Department of Microbiology, Immunology and Genetics UNT Health Science Center, Fort Worth, TX, USA; ^3^ Texas College of Osteopathic Medicine, Fort Worth, TX, USA; ^4^ Icahn School of Medicine at Mount Sinai, New York, NY, USA

## Abstract

**Background:**

Among the leading causes of mortality and morbidity worldwide, Alzheimer's disease (AD) and cancer both pose significant health challenges. AD is characterized by the buildup of neurofibrillary tangles and amyloid plaque, while cancer is characterized by unregulated proliferation and persistence of genetically modified cell. Multiple investigations have shown a negative association between several cancer types and AD, indicating that cancer patients have a lower likelihood of getting AD, whereas those diagnosed with AD have a reduced prevalence of cancer. To understand possible genetic underpinnings for the inverse association, we aim to identify local genetic correlation (rg) between AD and cancer (lung, breast, prostate and colorectal) by leveraging large‐scale genome‐wide association studies (GWAS) data.

**Method:**

We used GWAS of lung cancer [N_cases_=29,266], and meta‐analyzed GWAS summary statistics for late‐onset AD from two cohorts, Finnish biobank (FinnGen) G6_ALZHEIMER and Kunkle et al. [N_cases_=35,946 using METAL. We assessed the observed‐scale SNP heritability and genome‐wide genetic correlation (rg) using LD score regression (LDSC). Subsequently, we applied LAVA (Local Analysis of Variance and Association) to assess heritability in 2495 LD independent regions/loci. Significantly heritable regions in both AD and lung cancer were followed up for assessment of local rg.

**Result:**

We found insignificant global genetic correlation between AD and lung cancer rg=2.48% (*p* = 0.7168). We found negative local rg between AD and lung cancer in several positions for chromosome 6 and 19. In chromosome 6, we found local rg at chr6:27261036‐28666364=‐85.25% (*p* = 0.0019), chr6:31320269‐31427209=‐75.41% (*p* = 0.0126), chr6:31427210‐32208901=‐45.13% (*p* = 0.0244), chr6:32586785‐32629239=‐49.06% (*p* = 0.0108), chr6:32629240‐32682213=‐100% (*p* = 0.0175) and in chromosome 19:45040933‐45893307, rg=‐35.39% (*p* =  0.0046).

**Conclusion:**

We identified the APOE region on chromosome 19, and several regions near and within the MHC locus that exhibit negative correlations between AD and lung cancer. These findings suggest that APOE‐mediated mechanisms may play a role in the opposing biological processes underlying the two conditions. Additionally, the negative correlations observed in the MHC regions point to variability in immune function as a likely contributing mechanism. Our future analyses will include additional cancers, and the role of genetically regulated gene expression in these regions as potential mechanism underlying inverse association between AD and cancer.

